# Relationships between Biomarkers of Oxidative Stress in Seminal Plasma and Sperm Motility in Bulls before and after Cryopreservation

**DOI:** 10.3390/ani12192534

**Published:** 2022-09-22

**Authors:** Veronica Vigolo, Elisa Giaretta, Laura Da Dalt, Jana Damiani, Gianfranco Gabai, Federica Bertuzzo, Maria Elena Falomo

**Affiliations:** 1 Department of Animal Medicine, Production and Health (MAPS), Università di Padova, Viale dell’Università 16, 35020 Legnaro, PD, Italy; 2 Department of Comparative Biomedicine and Food Science, University of Padua, 35020 Legnaro, PD, Italy; 3 Intermizoo S.p.A, Via Dossetto 1, 30021 Caorle, VE, Italy

**Keywords:** AOPP, thiols, seminal plasma, sperm, bull

## Abstract

**Simple Summary:**

The early detection of (in)fertility biomarkers is of great interest in the enhancement of artificial insemination programs, with the objective of providing equal access to food resources and potentially improving conservation strategies for endangered species. (In)Fertility is a complex trait governed by a multitude of physiological, molecular, and external factors, and it has low heritability. Accurately assessing fertility markers in vitro and predicting reproduction outcomes in vivo is thus a difficult task. The current study aimed at evaluating the relationship between sperm motility parameters and oxidative stress (OS) markers of seminal plasma (SP) in bulls before and after cryopreservation. In this study, advanced oxidative protein products (AOPP) and thiol concentrations were found to be positively related to the higher motility of fresh ejaculates; on the other hand, they did not affect the motility parameters of frozen–thawed semen. These observations give an alternative perspective on the relationship between sperm motility and SP composition, in which increased OS in SP does not necessarily negatively affect sperm motility. There are still gaps in the knowledge about the effect of cryopreservation on sperm quality and the role of SP in the process. Nevertheless, sperm motility and OS parameters (AOPP and thiols) could be considered as effective biomarkers of bull (in)fertility.

**Abstract:**

This study aimed at evaluating the relationship between biomarkers of oxidative stress (OS) in seminal plasma and sperm motility in bulls before and after cryopreservation. Three ejaculates per bull were collected from 20 young bulls. Each ejaculate was analyzed for motility before and after cryopreservation (by CASA), and the SP concentration of Advanced Oxidation Protein Products (AOPP), thiols, and carbonyl groups (CT) were examined. Then, based on their motility, the ejaculates were grouped into: high motility fresh (HMF), low motility fresh (LMF), high motility thawed (HMT), and low motility thawed (LMT) groups. Higher AOPP and thiol concentrations on SP were related (*p* < 0.05) to the higher LIN and BCF and lower ALH of fresh semen. In addition, AOPP and thiols were significantly higher in HMF than LMF. As a confirmation of this, the Receiver Operating Characteristic (ROC) curve analysis showed that AOPP and thiol concentrations in SP were able to discriminate between HMF and LMF ejaculates (Area Under the Curve of 71.67% and 72.04%, respectively). These observations give an alternative perspective on the relationship between sperm motility and the OS parameters of SP, which need further investigations.

## 1. Introduction

Central testing is used to select young bulls that could produce the next generation of bulls in order to increase the net income of commercial cattle herds [1]. In the bovine artificial insemination (AI) industry, semen from young and genomic bulls is collected and marketed globally. The conventional analyses routinely performed on bull semen give important information on volume, sperm morphology, concentration, and motility; however, despite the adequate motility and morphological quality of the ejaculates, there is wide individual bull variability in fertilizing ability, whose molecular mechanisms are not known [2,3,4]. The prediction of in vivo reproductive performance is currently a challenge, and the relationship between fertility and semen components is one of the topics in current andrology research.

Many recent studies are focused on seminal plasma composition, investigated with OMIC approaches such as metabolomics [5,6,7,8] and proteomics [9,10]. The evaluation of oxidative status markers (OSM) and their relation with fertility is an important task as well since it helps to better understand spermatogenesis, predict male fertility, and improve semen quality after storage for AI success [11]. Reactive oxygen species (ROS) include highly reactive free radicals that allow sperm capacitation, hyperactivation, and acrosomal reaction in low doses, but their excessive levels lead to lipid peroxidation (LPO), with the consequent loss of sperm motility, membrane integrity, and increased DNA damage [12,13,14]. The correct balance between ROS and antioxidants is fundamental for the maintenance of semen quality. Seminal plasma components such as proteins, ROS inhibitors, and antioxidants play a leading role in sperm protection. For this reason, the removal of seminal plasma in bulls during the cryopreservation procedure is still contradictory, although it has been demonstrated that its presence causes a reduction in sperm motility [15,16].

Antioxidant compounds are produced under stressful conditions and are used as effective neutralizers against ROS. There is a network of several antioxidants as well as enzymatic, non-enzymatic, and low molecular weight compounds that work together to maintain prooxidant–antioxidant homeostasis within cells [17]. Part of these important antioxidants are thiols, a group of cysteine residues containing sulfhydryl groups (–SH). This large class of antioxidants includes cysteine, N-acetyl cysteine (NAC), and glutathione (GSH) [18]. Free –SH groups are typically highly conserved in proteins, where cysteine amino acids act as stabilizing, catalytic, metal-binding, and redox-regulatory entities [19]. Intracellular thiol concentrations have been found to be strongly correlated with sperm motility parameters post-thaw. Additionally, studies report a depletion of intracellular thiols after thawing and an increase in sperm senescence [18,20]. The increased oxidation of these groups may lead to an excessive accumulation of oxidative modified proteins [21,22].

Protein carbonyl groups are effective biomarkers of OS because of their stability and early formation. Their levels have been proven to be higher in low-quality semen in men and bovine ejaculates, together with higher ROS levels [23,24]. These findings were further supported and further developed by Mostek et al. in 2018 [24], who established a correlation between levels of reactive oxygen species, protein carbonylation, and sperm quality. This study showed that the increasing levels of reactive oxygen species are linked to an increase in protein carbonylation and thus poorer sperm quality.

Other OS markers such as advanced oxidation protein products (AOPP) have also been shown to be crucial components in characterizing the OS damage. Advanced oxidation protein products are oxidative markers associated with the damage of plasma proteins [25,26]. Although recent studies have demonstrated that increased ROS in men SP were related to higher levels of AOPP and men infertility, no study using AOPP as OS biomarkers has so far been conducted to test animal semen [17,27].

The aim of this study is to investigate the relation between sperm motility and the seminal plasma concentrations of AOPP, thiols, and protein carbonyl groups in bull semen before and after cryopreservation.

## 2. Materials and Methods

A short scheme representing the experimental approach is presented in the Supplementary File (Figure S1).

### 2.1. Semen Collection and Preparation

Semen samples were obtained from 20 young bulls aged from 10 to 12 months and housed at the Intermizoo A.I. station in the Vallevecchia farm in Brussa, Carole (Venice, Italy). Semen was collected using an artificial vagina and was diluted 1:1 with the pre-warmed (+37 °C) commercial extender. Seminal plasma (obtained after centrifugation of an aliquot of each ejaculate) was stored at −80 °C for analyses, as described below. After dilution and collection of the SP aliquot, the semen samples were immediately cryopreserved. The diluted semen samples were packed into 0.5 mL labelled plastic straws (50–80 × 10^6^ spermatozoa/mL). This procedure was performed at 4 °C. Afterward, the straws were transferred to a programmable freezer. The freezing program consisted of the following rates: −4 °C/min from 4 °C to 0 °C, −1 °C/min from 0 °C to −4 °C, −12 °C/min from −4 °C to −40 °C, −30 °C/min from −40 °C to −140 °C. The straws were finally plunged into liquid N_2_ (at −196 °C) for further storage.

### 2.2. Sample Evaluation

Sperm samples were assessed for motility by a computer-assisted sperm analysis system (CASA, Hamilton Thorne, IVOS Ver. 12) before and after the freezing–thawing protocol, using the standard bull setup (60 frames per second; min contrast, 35 min cell size, 8 pixels; progressive cells, VAP ≥25.0 µm/s; straightness, ≥75%; static cell cutoff, VAP = 24.9 µm/s, VSL 20.0 µm/s). Approximately one thousand sperm cells for each sample diluted at 30 × 10^6^ sperm/mL were captured and evaluated using a fixed-height Leja Chamber SC 20-01-04-B (Leja, The Netherlands). Sperm quality parameters were assessed on the same ejaculates, both as fresh (FR) samples and after thawing (TH), and so were total motility (TM, %), progressive motility (PM, %), curvilinear velocity (VCL, mm/s), average path velocity (VAP, mm/s), straight-line velocity (VSL, mm/s), straightness (STR, %), linearity (LIN, %), average lateral head displacement (ALH, mm), and beat cross frequency (BCF, Hz). Total motility was defined as the percentage of spermatozoa that showed an average path velocity greater than 25 µm/s and a straight path velocity higher than 20 µ/s, whereas progressive motility was defined as the percentage of spermatozoa that showed an average path velocity greater than 25 µm/s and a straightness greater than 75%.

Protein concentrations in SP were measured by the BCA method (BCA Protein assay kit; Pierce Biotechnology, Rockford, IL, USA), following the manufacturer’s instructions. Carbonyl groups and AOPP concentrations were measured as biomarkers of protein oxidation in SP. The AOPP concentration was measured spectrophotometrically [28]. The samples (0.2 mL diluted 1:2 in 20 mM phosphate buffer, pH 7.4) were placed in 96-well microplates (Perkin–Elmer Life Analytical Sciences, Shelton, CT, USA) and mixed with 20 µL of acetic acid. A standard curve was prepared using 0.2 mL of a chloramine T solution (0–100 µmol/L; Sigma–Aldrich, St. Louis, MO, USA) as the reference, along with 10 µL of 1.16 M potassium iodide and 20 µL of acetic acid. The absorbance of the reaction mixtures was immediately read at 340 nm in a microplate reader (Victor X4 2030 Multilabel Reader, Perkin-Elmer, Waltham, MA, USA) against blank (0.2 mL of 20 mM phosphate buffer, 10 µL of 1.16 M potassium iodide, and 20 µL of acetic acid). The AOPP content was expressed as chloramine T equivalents.

Protein carbonyls were measured following derivatization with DNPH (2,4 dinitrophenylhydrazine; Sigma-Aldrich Co., St. Louis, MO, USA). Samples of SP (100 µL) were divided into two aliquots; one intended for use as negative control and the other for DNPH derivatization. After a first precipitation of the proteins with 10% trichloroacetic acid, 0.5 mL of 10 mM DNPH solubilized in 2.5 N HCl were added in the series of samples to be derivatized and 0.5 mL of 2.5 N HCl respectively in the negative controls. After a second precipitation of proteins with 20% trichloroacetic acid, the supernatant was removed, and the pellet was re-solubilized with 1 mL of ethanol/ethyl acetate (1:1 *v*:*v*) and centrifuged (4 °C, for 10 min at 5000× *g*) 3 times to remove excess DNPH. After washing, 1 mL of Guanidine-HCl 6 M was added, and the samples were incubated at 37 °C for 15 min. The carbonyl content was determined by reading the absorbance at 380 nm with a spectrophotometer (V 630, Jasco Europe, Cremella, LC, Italy), using the molar extinction coefficient of DNPH (ε = 22,000 M^−1^ cm^−1^) [29]. The carbonyl content was expressed as nmol/mL SP.

Thiol concentration in SP (nmol/mL) was determined according to the thiol/disulfide reaction using Ellman’s reagent [30]. Sulfhydryl groups were estimated using a standard curve prepared by serial dilutions of cysteine standards (Sigma), from 0.25 to 1.5 mM. A total of 200 µL of each cysteine dilution or 200 µL of SP samples (diluted 1:10) were added to the microtiter plate wells. Thiol concentration was measured by reading the absorbance at 412 nm after the addition of 3.5 µL of Ellman’s reagent (Sigma), which was expressed as nmol/mL SP.

### 2.3. Statistical Analysis

Data were analyzed using the R statistical environment v. 3.6.2 (The R Foundation for Statistical Computing, Vienna, Austria). Sperm motility parameters (VAP, VSL, VCL, ALH, BCF, TM, and PM), measured in both FR and TH samples, underwent principal component analyses (PCA). Then, the first three principal components (PCs), which explained more than 90% of the variance, were used to perform cluster analyses on both FR and TH samples. The k-means method was used to perform the cluster analysis. As a result, two clusters were obtained for the FR samples—named high motility fresh (HMF) and low motility fresh (LMF) groups, and two clusters were obtained for the TH samples—named high motility thawed (HMT) and low motility thawed (LMT) groups. As for the fresh samples, 44 of them belonged to the HMF group, while the other 16 belonged to the LMF group. Concerning the thawed samples, 39 of them belonged to the HMT group, and the other 21 belonged to the LMT group.

Linear mixed models were used to investigate the relationship between the motile parameters of FR and TH semen and the respective clusters obtained. For each model, the fixed effect was cluster, and the random effect was bull. In all cases, each motility sperm parameter (TM, PM, VAP, VCL, VSL, STR, LIN, ALH, and BCF) was the dependent variable. In addition, general linear models were used to study the relationship among the OS parameters (AOPP, Thiols, and Carbonyls) and the fresh (HMF and LMF) and thawed (HMT and LMT) groups. For these models, the OS parameters were considered as the fixed effects, while the fresh (levels: HMF and LMF) or frozen (levels: HMT and LMF) clusters were the dependent variables. The binomial family was used in the model.

Linear models were utilized to investigate the relation between the motility parameters of FR and TH semen and the OS parameters of seminal plasma. In all cases, each motility sperm parameter was the dependent variable, and the OS parameter of seminal plasma was the fixed factor. For each model, the residues were checked for variance homogeneity and normal distribution. An ROC analysis was used to determine the AUC of the oxidative status parameters, which significantly affect semen clusterization. For these oxidative status parameters, the best cut-off, the related sensitivity and specificity, and the AUC were recorded.

Finally, the correlation between motility variables and OS parameters were determined by Spearman test for non-parametric variables. In all statistical analyses, the minimal level of significance was set at *p* < 0.05. Data are expressed as mean ± sd.

## 3. Results

### 3.1. Relation between Groups and Motility Parameters

In fresh semen, all the motility parameters studied showed significant differences between the HMF and LMF groups. More specifically, TM, PM, VAP, VCL, VSL, LIN, BCF, and STR were significantly higher in the HMF group as compared to the LMF group (Table 1). Otherwise, in the same cluster, ALH was significantly lower (*p* < 0.05) in the HMF group as compared to the LMF group (Table 1). In frozen semen, motility variables TM, PM, ALH, VAP, VCL, and VSL presented significantly higher values in the HMT group as compared with the LMT group (Table 1).

### 3.2. Relation between Groups and OS Parameters of Seminal Plasma

**Figure 1:**
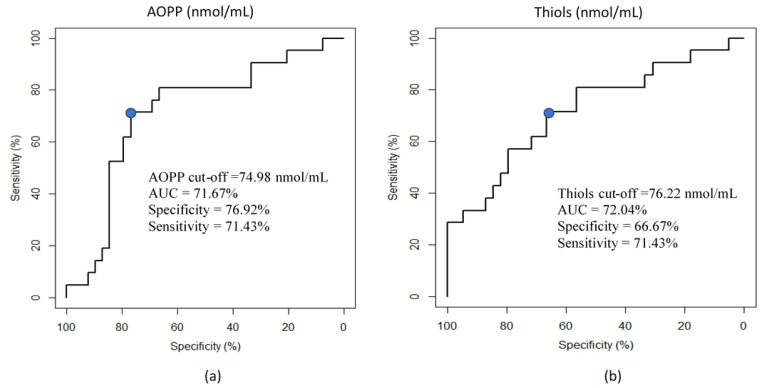
Receiver operating characteristic (ROC) curve analysis for the prediction of motility clusters. (**a**) ROC curve showing the ability of the AOPP concentration (nmol/mL) of seminal plasma (SP) to discriminate the motility of fresh semen (HMF vs. LMF). AUC: area under the curve. The blue dot shows the cut-off of AOPP to predict the motility group (HMF vs. LMF) (**b**) ROC curve showing the ability of the thiol concentration (nmol/mL) of seminal plasma (SP) to discriminate the motility of fresh semen (HMF vs. LMF). AUC: area under the curve. The blue dot shows the cut-off of thiols to predict the motility group (HMF vs. LMF).

AOPP and thiols were significantly higher in the HMF group as compared with the LMF group (Table 2). As reported in Figure 1, the ROC curve analysis shows that AOPP (nmol/mL) and Thiol (nmol/mL) concentrations were able to discriminate between the HMF and LMF ejaculates, thus showing a fair, discriminant value with an AUC of 71.67% and 72.04%, respectively. No significant differences were observed in the OS parameters within the TS groups. Significant positive correlations were found between AOPP and carbonyls (r = 0.78, *p* < 0.01), between AOPP and thiols (r = 0.88, *p* < 0.01), and between carbonyls and thiols (r = 0.86, *p* < 0.01).

### 3.3. Relation between Motility and OS Parameters of Seminal Plasma

**Figure 2:**
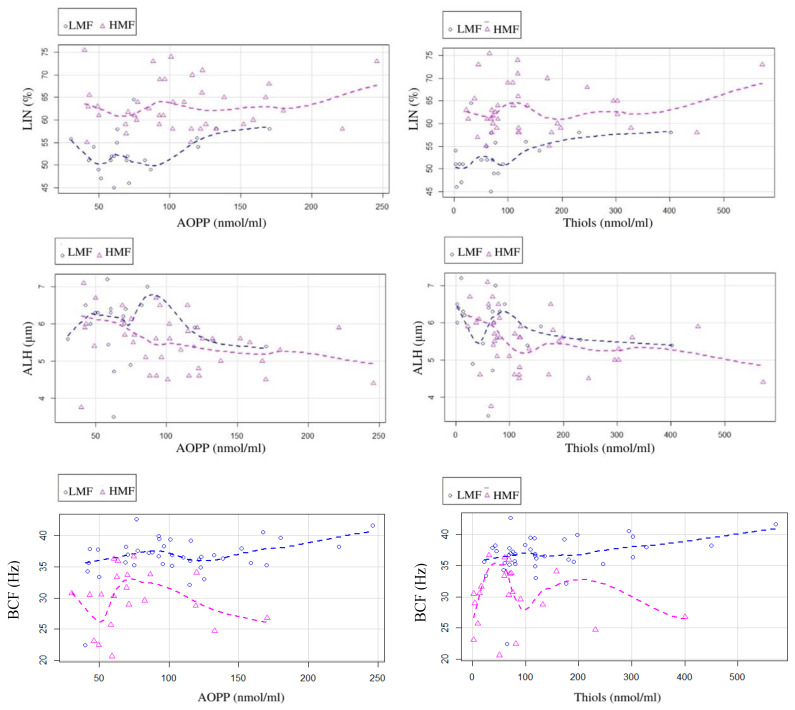
Scatter plot representing the relation among the oxidative stress markers, AOPP and thiols, of seminal plasma and the motility parameters, LIN, ALH, and BCF.

Significant negative correlations with AOPP (r = −0.403, *p* < 0.05) and thiols (r = −0.42, *p* < 0.05) were observed for the ALH of fresh semen (Figure 2). Weak positive correlations with AOPP (LIN: r = 0.35, *p* < 0.05; BCF: r = 0.37, *p* < 0.05) and thiols (LIN: r = 0.33, *p* < 0.05; BCF: (r = 0.35, *p* < 0.05) were detected for the LIN and BCF of fresh semen (Figure 2). No significant correlations were observed between the motility and OS parameters of frozen semen.

General linear models highlighted the significant (*p* < 0.05) influence of AOPP and thiols concentrations in SP on the LIN, BCF, and ALH of fresh semen. In particular, LIN and BCF were positively influenced by the increase in AOPP and thiols in SP, while these OS markers showed a negative impact (*p* < 0.05) on ALH. Conversely, a positive weak correlation (r = 0.33; *p* < 0.05) was observed between BCF and the thiols in the HMF group, but not in the LMF group. A similar picture was observed between BCF and SP AOPP concentration (r = 0.37; *p* > 0.01; Figure 2).

## 4. Discussion

Oxidative stress is the cause of a series of pathological conditions that involve different apparatus, including the reproductive system, for which reason it may lead to male and female infertility [31,32,33]. In our study, bull semen samples were divided into two groups with the use of a cluster analysis, according to motility parameters. The obtained HMF and HMT groups had higher motility parameters as compared to the LMF and LMT groups, respectively [34].

According to our analyses of the OS parameters, we could hypothesize that AOPP and thiol levels in seminal plasma could be predictive of fresh semen motility. In fact, significantly higher concentrations of AOPP and thiols were observed in the SP of the HMF group in comparison with that of the LMF group. This relationship was also confirmed by the results of the ROC curves. Although excessive ROS production is usually associated with mitochondrial dysfunction, DNA alterations, membrane permeability, and motility impairments, minimum levels of ROS are required for hyperactivation, capacitation, and oocyte–sperm fusion [14]. Several studies have investigated the relationship between OS and male fertility, assessing various OS markers in SP with the use of different techniques such as the quantification of ROS levels by chemiluminescence and the measurement of the oxidation reduction potential by electrochemical assay [35,36]. While some studies have analyzed the intracellular concentration of thiols and carbonylated proteins, no study has so far evaluated the concentrations of AOPP and thiols in SP in relation to animal reproduction. In fact, a limitation of bull semen is its lower amount of seminal plasma as compared to that in swine and equine species, which does not allow for the carrying out of more in-depth evaluations. Moreover, the fertility parameters considered and related to the OS in SP are also different between studies: sperm viability, morphology, motility, and DNA fragmentation are the most relevant assessment used to provide insight into spermatozoa quality and fertilizing ability [35,36,37,38,39]. As regards semen motility, the use of different settings and instruments should be taken into account when comparing with other studies. The frame rate of 60 frames per second was used in this work, as reported in recent previous studies and in in accordance with the Standard Operating Procedure of Italian Experimental Institute “Lazzaro Spallanzani” (Law D.M. 403/2000, 2013) [2,39,40]. However, a more accurate assessment of the effects of cryoconservation on the alteration of the sperm physiology could be achieved by using higher acquisition rates of sperm motility (e.g., up to 200 fpr), which is suggested for fast nonlinear sperm such as that of the bull [41]. The use of higher frame rates, as suggested by other authors for semen motility analysis [41,42], is difficult to obtain with commercial CASA, for which the maximum settable value is 60 fps. As reported by [42], STR and LIN are significantly affected by the frame rate setting, which consequently determines different PM results. Indeed, the comparison of motility results obtained by different studies is limited by the use of different settings and tools.

In contrast with our results, ref. [27] observed increased concentrations of AOPP in the SP of azoospermic and theratozoospermic patients versus fertile men. In the same study, a significant decrease in melatonin, which acts as an antioxidant, was observed in infertile subjects. On the other hand, in our study, significantly higher concentrations of AOPP and thiols were observed in the SP of HMF semen. However, while the energy metabolism of human semen is dependent on glycolytic pathways [43], in other species such as equine and bovine, the main generation of ATP is reliant on intensive mitochondrial activity through oxidative phosphorylation [44,45,46,47]. In bull semen, the regulation of energy metabolism for the maintenance of sperm motility is not yet fully understood. Although previous studies on bovine sperm observed that anaerobic glycolytic metabolism could be enough to maintain active motility, a more recent investigation found that higher mitochondrial and metabolic functions are linked to higher total motility [47]. In addition, a recent study by [46], partially in agreement with [47], confirmed mitochondrial participation in the regulation of bovine sperm motility, even though the use of specific mitochondrial complex inhibitors did not affect sperm motility, which was maintained by the glycolytic ATP production. Thus, considering that semen motility is dependent on energy production, a more intense mitochondrial activity in higher motility bull ejaculates could determine an increase in ROS production and consequently greater protein oxidation in SP, as suggested by the higher concentration of AOPP. This paradoxical association between oxidative stress, motility, and fertility has already been found in horse semen by the authors of [44,48].

Thiols are known to be efficient antioxidants, and their high concentrations are indicative of low levels of OS [49,50]. The increased oxidation of thiol groups due to increased ROS can cause a general alteration of the thiol–disulfide status, with the consequent reduction of the total antioxidant capacity of SP [22]. In our study, higher levels of AOPP are accompanied by higher thiol concentrations in SP, and both are higher in the HMF semen. These observations are what we physiologically expected and may be interpreted as the result of a better balance between pro-oxidant and antioxidant molecules in high-motility semen. Even though the general assumption on OS is that it is regulated by an even production of antioxidants, numerous studies give different explanations for the interactions and relationship between the pro-oxidant and antioxidant components [27,51]. However, the positive correlation found between AOPP and thiol concentrations might not be enough to assume a causal relationship, where the increased levels of antioxidants are a response of the increase in protein oxidation [27]. Indeed, the damage caused by ROS could be irreversible, regardless of the amount of antioxidants secreted, making it a tedious task to accurately assess sperm quality and fertility capacities. For this reason several studies have shown the role of supplementation of different antioxidants, such as melatonin, resveratrol, and catalase, on the freezing and/or thawing media [52,53,54].

It is interesting that in our study, no difference in the oxidative status parameters of seminal plasma has been observed between high and low motility groups in frozen–thawed semen (HMT and LMT groups). To the best of our knowledge, no studies have so far elucidated the energy metabolism of thawed bull semen, in which different metabolic pathways could probably be found as compared to fresh semen.

In this study, AOPP and thiol concentrations in fresh semen samples were negatively correlated with ALH and positively correlated with LIN and BCF. Higher ALH, exhibited by hyperactivated spermatozoa, is considered to be a marker of sub-fertility in bull ejaculates [34,55]. Indeed, the presence of hyperactivated spermatozoa in the ejaculate is an index of premature capacitation [56,57,58]. While the same trend was observed in the HMF and LMF groups for ALH and LIN, a significantly different relationship was found between BCF and the motility groups. BCF was positively correlated with AOPP and thiols in the HMF group, confirming that the great amount of ATP production in spermatozoa, which exhibited the higher frequency of flagellar beats, could lead to higher mitochondrial oxidative metabolism, and thus to greater AOPP production, buffered by higher thiol concentration in SP. These results provide evidence that OS parameters can be considered as predictive for sperm kinetics as well.

Although mitochondrial activity was not evaluated in this study, further evaluation of semen could investigate if the intense mitochondrial activity of the HMF semen is the main source of ROS, which would result in a higher amount of AOPP, counteracted by higher concentrations of thiols in SP. Based on these observations, it is assumed that seminal plasma may have an important, protective function against ROS leakage from active sperms. However, in this experiment, we could not identify which specific proteins and metabolites are involved in this phenomenon; more in-depth studies on metabolomics and proteomics of SP could be useful for identifying new biomarkers of OS. In addition, the monitoring of spermatozoa ROS production through the detection of intracellular markers, such as membrane lipid peroxidation as well as intracellular superoxide and peroxide production could be related to the SP parameters [59].

## 5. Conclusions

Bull fertility is a multifactorial trait, with low heritability and a large variation between individuals and species. This study demonstrated the importance of evaluating the composition of SP in relation to sperm motility and cryopreservation. As SP has both inhibitory and stimulatory roles in sperm function and during cryopreservation, the identification of SP components that may play a critical role in determining sperm quality could be beneficial for the prediction of semen fertility. Parameters of the OS could be efficient fertility biomarkers in bulls. However, further studies are required to understand the causal relationship between sperm motility and OS. A deeper investigation of SP composition by OMIC approaches would help to better understand and predict bull fertility and freezability.

## Figures and Tables

**Table 1:** Motility parameters of fresh (FR) and frozen–thawed (FT) semen in high-motility and low-motility clusters (respectively HMF and LMF for FR semen and HMT and LMT for TH semen).

Fresh Semen			Frozen–Thawed Semen		
Variable	Cluster	Mean ± SD	Variable	Cluster	Mean ± SD
TM, %	LMF	67.89 ± 17.40 *	TM, %	LMT	43.03 ± 17.23 *
	HMF	87.34 ± 7.41		HMT	76.55 ± 8.98
PM, %	LMF	50.36 ± 12.32 *	PM, %	LMT	35.58 ± 14.12 *
	HMF	68.53 ± 6.36		HMT	64.7 ± 8.67
VAP, μm/s	LMF	97.75 ± 13.85 *	VAP, μm/s	LMT	73.23 ± 10.45 *
	HMF	124.12 ± 14.6		HMT	100.68 ± 8.9
VCL, μm/s	LMF	161.52 ± 24.67 *	VCL, μm/s	LMT	121.92 ± 18.38 *
	HMF	175.83 ± 23.52		HMT	151.7 ± 17.74
VSL, μm/s	LMF	80.05 ± 10.44 *	VSL, μm/s	LMT	62.39 ± 7.97 *
	HMF	107.85 ± 12.43		HMT	87.36 ± 7.93
STR, %	LMF	82.12 ± 3.07 *	STR, %	LMT	86.04 ± 3.29
	HMF	85.8 ± 2.76		HMT	86.83 ± 3.11
LIN, %	LMF	52.82 ± 4.63	LIN, %	LMT	54.6 ± 3.69
	HMF	63.21 ± 5.38		HMT	60.01 ± 6.28
ALH, μm	LMF	5.88 ± 0.83	ALH, μm	LMT	4.93 ± 0.78
	HMF	5.5 ± 0.75		HMT	5.64 ± 0.76
BCF, Hz	LMF	29.93 ± 6.64 *	BCF, Hz	LMT	27.69 ± 4.93
	HMF	36.67 ± 3.23		HMT	26.15 ± 3.23

Asterisks indicate significantly different means (*p* < 0.05) for each motility variable between the LMF and HMF and the LMT and HMT groups within the same column.

**Table 2:** Oxidative stress markers in seminal plasma of fresh and frozen–thawed semen in high-motility and low-motility clusters (respectively HMF and LMF for fresh semen and HMT and LMT for thawed semen).

Fresh Semen					Frozen–Thawed Semen				
Variable	Cluster	Mean ± SD	Median	IQR	Variable	Cluster	Mean ± SD	Median	IQR
AOPP, nmol/mL	LMT	75.36 ± 33.86 *	1.29	24.22	AOPP, nmol/mL	LMT	94.29 ± 48.16	69.97	67.09
	HMF	106.9 ± 47.42	1.54	48.40		HMF	96.43 ± 45.01	92.55	47.77
CT, nmol/mL	LMT	6.57 ± 6.46	4.91	3.34	CT, nmol/mL	LMT	7.55 ± 7.39	5.47	3.32
	HMF	8.44 ± 6.08	6.23	7.76		HMF	7.87 ± 5.84	5.71	5.75
Thiols, nmol/mL	LMT	81.07 ± 92.36 *	68.16	64.96	Thiols, nmol/mL	LMT	121.46 ± 114.06	1.17	2.63
	HMF	143.74 ± 118.6	109.67	108.77		HMF	121.93 ± 114.48	1.58	1.05

Asterisks indicate significantly different means (*p* < 0.05) for each OS variable between the LMF and HMF and the LMT and HMT groups within the same column.

## Data Availability

Data is contained within the article or Supplementary Material.

## Supplementary Materials

The following supporting information can be downloaded at: https://www.mdpi.com/article/10.3390/ani12192534/s1, Figure S1: Scheme representing the experimental approach.
