# Supplementary figures and images for: Relationships between Biomarkers of Oxidative Stress in Seminal Plasma and Sperm Motility in Bulls before and after Cryopreservation
Source: Animals (Basel). 2022 Sep 22;12(19):2534. doi: 10.3390/ani12192534 (PMC9558952; doi:10.3390/ani12192534)

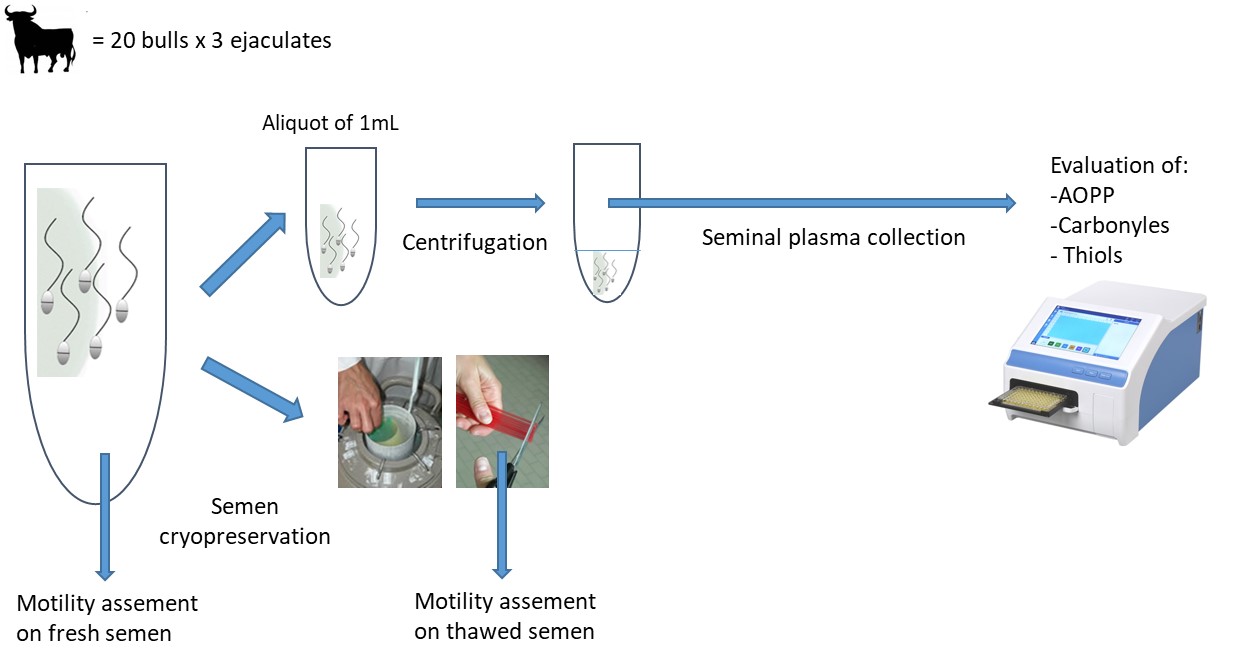

Supplement: Supplementary file 1 [file animals-12-02534-s001.zip › animals-1904583-supplementary Figure S1.jpg]
